# Characterization of probiotic *Escherichia coli *isolates with a novel pan-genome microarray

**DOI:** 10.1186/gb-2007-8-12-r267

**Published:** 2007-12-18

**Authors:** Hanni Willenbrock, Peter F Hallin, Trudy M Wassenaar, David W Ussery

**Affiliations:** 1 Center for Biological Sequence Analysis, Technical University of Denmark, 2800, Lyngby, Denmark; 2 Exiqon A/S, 2950 Vedbæk, Denmark; 3 Molecular Microbiology and Genomics Consultants, Tannenstrasse, 55576 Zotzenheim, Germany

## Abstract

**Background:**

Microarrays have recently emerged as a novel procedure to evaluate the genetic content of bacterial species. So far, microarrays have mostly covered single or few strains from the same species. However, with cheaper high-throughput sequencing techniques emerging, multiple strains of the same species are rapidly becoming available, allowing for the definition and characterization of a whole species as a population of genomes - the 'pan-genome'.

**Results:**

Using 32 *Escherichia coli *and *Shigella *genome sequences we estimate the pan- and core genome of the species. We designed a high-density microarray in order to provide a tool for characterization of the *E. coli *pan-genome. Technical performance of this pan-genome microarray based on control strain samples (*E. coli *K-12 and O157:H7) demonstrated a high sensitivity and relatively low false positive rate. A single-channel analysis approach is robust while allowing the possibility for deriving presence/absence predictions for any gene included on our pan-genome microarray. Moreover, the array was highly sufficient to investigate the gene content of non-pathogenic isolates, despite the strong bias towards pathogenic *E. coli *strains that have been sequenced so far.

**Conclusion:**

This high-density microarray provides an excellent tool for characterizing the genetic makeup of unknown *E. coli *strains and can also deliver insights into phylogenetic relationships. Its design poses a considerably larger challenge and involves different considerations than the design of single strain microarrays. Here, lessons learned and future directions will be discussed in order to optimize design of microarrays targeting entire pan-genomes.

## Abstract 2

A high-density microarray has been designed that covers the genomes of 24 Escherichia coli and 8 Shigella strains. As a proof-of-principle the genomes of four probiotic E. coli strains were analyzed and their phylogenetic relationship to other E.coli strains investigated.

## Background

Bacterial isolates are traditionally classified into species by bacteriological methods, and subtyped within the species by phenotypic or genotypic characterization. For the identification and subtyping of *Escherichia coli *isolates, a wide variety of typing methods have been developed. A recent addition to this spectrum is array comparative genomic hybridization (aCGH) [1]. Thus, microarray hybridization is becoming a standard procedure to evaluate the genetic content of a bacterial species. For *E. coli*, a microarray covering the gene content of seven strains was recently developed for the characterization of emerging pathogens [2]. However, since then, many additional *E. coli *strains and plasmids have been sequenced, and the total number of genes potentially present in *E. coli *strains, the so-called 'pan-genome' [3,4], increases with each new *E. coli *genome sequenced. A microarray chip approximating the complete pan-genome of *E. coli *would provide optimal sensitivity to characterize isolates. Here, we present a novel design of a microarray covering the complete currently known genome content of 32 sequenced genomes. Such a pan-genome microarray can be used for more precise characterization of novel strains, including emerging pathogens, and can also deliver insights into phylogenetic relationships.

Phylogenetic relationships are commonly determined by bacterial subtyping. Due to the complex sexual behavior of bacteria, phylogenetic trees obtained with individual genes often do not correspond to each other. Although multilocus sequence typing is now regarded by many as a good standard to determine phylogenetic relationships between and within bacterial species, it does not always reflect the true genetic diversity of members of a species; trees based on multilocus sequence typing may, therefore, differ significantly from a tree based on whole gene content [3]. A pan-genome microarray may offer a suitable alternative to complete genome sequencing for extracting the necessary gene content to construct a realistic phylogenetic tree based on conserved gene content. The recent technological development in sequencing and the consequent price drop have led to an explosion of available genome sequences and perhaps within a few years will lead to sequencing being a faster and cost effective alternative to CGH microarray analysis. However, at the moment, sequencing is still more costly and less time efficient than hybridization experiments, while hybridization experiments potentially also can provide information regarding gene expression.

Here, we determine an approximate *E. coli *pan-genome, based on 24 *E. coli *and 8 *Shigella *genomes available at the time of analysis (November 2006). The inclusion of *Shigella *genomes was justified as the genus division between *Shigella *and *Escherichia *is historical but taxonomically incorrect [5,6]. For simplicity, the *Shigella *and *E. coli *genomes are collectively referred to as *E. coli*. From these genomes we construct an *E. coli *pan-genome microarray. The technical performance of this pan-genome microarray is assessed by the correct identification of present and absent genes from the completely sequenced genome of the MG1655 isolate of *E. coli *strain K-12 (hereafter referred to as MG1655) and strain O157:H7 EDL933 (EDL933 for short), collectively referred to as the control strains. Pathogenic *E. coli *isolates are highly overrepresented in the available genome sequences and, hence, on our pan-genome chip. We assessed whether this chip could nevertheless be useful for characterization of non-pathogenic isolates by hybridizing four probiotic *E. coli *isolates to the chip. These isolates are part of a commercially available product (Symbioflor2) marketed for human use as an enhancer of the immune system. The product contains viable bacteria comprising at least four genotypes of commensal *E. coli*. By characterizing their gene content, we investigated the phylogenetic relationship of these isolates to other *E. coli *strains.

## Results

### Defining the *E. coli *core-genome and pan-genome

**Figure 1:**
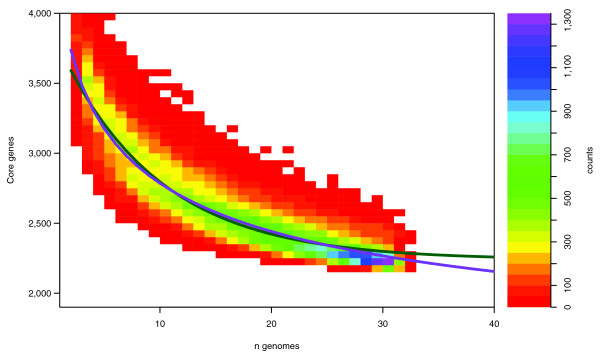
Two-dimensional density plot of 'core genes' for the *E. coli *pan-genome. The plot illustrates the number of *E. coli *core genes for *n *= 2,...,32 genomes based on a maximum of 3,200 random combinations of genomes for each n. The density colors reflect the count of combinations giving rise to a certain number of core genes; that is, for *n *= 3, genome number 3 is compared to genomes 1 and 2, and the number of core genes is the number of genome 3 genes conserved in genomes 1 and 2. The green line is the fit to the exponential decay function by [3], and the red line is our proposed fit to a slightly modified decay function as explained in the Materials and methods.

For each of the considered genome and plasmid sequences listed in Table 1, genes were predicted by EasyGene [7,8] and translated into proteins. These were considered conserved (belonging to the same protein gene group) if they showed a sequence similarity of 50% or higher along at least 50% of the full length of the protein sequence according to the similarity criteria defined in [3] (see Materials and methods for details). The core genome, that is, the number of conserved genes present in all genomes, was estimated by fitting an exponential decay function by non-linear least squares (Figure 1). In short, for each number of genomes (n), the gene content was compared for multiple random combinations of n genomes after which a best fit decay curve was fitted. Two slightly different decay functions were used: the originally suggested decay function based on [3] (Figure 1, green line) did not fit the data as well as a slightly modified exponential decay function (Figure 1, red line) (see Materials and methods for details on the applied modifications). Based on the best-fitting extrapolation, we estimate the size of the core genome to approach approximately 1,563 genes for an infinite (or very large) number of *E. coli *genomes.

**Table 1:** Sequences included in the microarray design

Strain	Accession	NCBI Proj ID	Contigs	ORFs	Length
*E. coli *042 chromosome	-*	340	1	4,607	5,241,977
*E. coli *042 plasmid	-	340	1	106	113,346
*E. coli *101-1 chromosome	AAMK01000001-70	16193	70	4,353	4,880,382
*E. coli *53638 chromosome	AAKB01000001-119	15639	119	4,779	5,289,471
*E. coli *536 chromosome	CP000247	16235	1	4,341	4,938,920
*E. coli *B chromosome	-	18083	1	4,076	4,629,819
*E. coli *B171 chromosome	AAJX01000001-159	15630	159	4,780	5,299,753
*E. coli *B171 plasmid	AB024946	15630	1	69	68,817
*E. coli *B7A chromosome	AAJT01000001-198	15572	198	4,646	5,202,558
*E. coli *CFT073 chromosome	AE014075	313	1	4,653	5,231,428
*E. coli *E11019 chromosome	AAJW01000001-15	15578	115	4,839	5,384,084
*E. coli *E22 chromosome	AAJV01000001-109	74230453	109	4,943	5,516,160
*E. coli *E2348 chromosome	-	341	4	4,592	5,071,653
*E. coli *E2348 pB171 plasmid	-	341	1	70	68,890
*E. coli *E2348 p9123 plasmid	-	341	1	5	6,293
*E. coli *E2348 pGEPAT plasmid	-	341	1	3	2,233
*E. coli *E24377A chromosome	AAJZ01000001	13960	1	4,407	4,980,187
*E. coli *F11 chromosome	AAJU01000001-88	15576	88	4,593	5,206,906
*E. coli *H10407 chromosome	-	-	89	4,865	5,428,706
*E. coli *HS chromosome	AAJY01000001	13959	1	4,126	4,643,538
*E. coli *K12-MG1655 chromosome	U00096	225	1	4,122	4,639,675
*E. coli *K12-W3110 chromosome	AP009048	16351	1	4,133	4,646,332
*E. coli *O103Oslo chromosome^†^	-	-	1115	4,571	5,231,845
*E. coli *O157RIMD0509952 chromosome	BA000007	226	1	4,989^‡^	5,498,450
*E. coli *O157RIMD0509952 pO157	AB011549	226	1	70	92,721
*E. coli *O157RIMD0509952 pOSAK1	AB011548	226	1	3	3,306
*E. coli *RS218 chromosome	-	-	1	4,898	5,089,234
*E. coli *RS218 plasmid	-	-	1	115	114,233
*E. coli *UTI189 chromosome	CP000243	16259	1	4,466	5,065,741
*E. coli *UTI189 plasmid	CP000244	16259	1	114	114,230
*E. coli *VR50 chromosome^†^	-	-	1228	4,453	5,064,870
*E. coli *APEC-O1 chromosome	CP000468	16718	1	4551	5,082,025
*E. coli *O157EDL933 chromosome	NC_002655	259	1	4,664^‡^	5,528,445
*E. coli *O157EDL933 plasmid	AF074613	259	1	70	92,077
*S. boydii *Sb227 chromosome	CP000036	13146	1	4,356	4,519,823
*S. dysenteriae *M131649 chromosome	-	346	234	4,755	4,962,690
*S. dysenteriae *Sd197 chromosome	CP000034	13145	1	4,237	4,369,232
*S. dysenteriae *Sd197 pSD1197	CP000035	13145	1	160	182,726
*S. flexneri *2457T chromosome	AE014073	408	1	4,388	4,599,354
*S. flexneri *301 chromosome	AE005674	310	1	4,410	4,607,203
*S. flexneri *301 pCP301 plasmid	AF386526	310	1	194	221,618
*S. flexneri *8401 chromosome	CP000266	166375	1	4,383	4,574,284
*S. sonnei *53G chromosome	-	-	5	4,780	5,220,473
*S. sonnei *Ss046 chromosome	CP000038	13151	1	4,443	4,825,265
*S. sonnei *Ss046 pSS plasmid	CP000039	13151	1	179	214,396

*In progress: the genome sequence has not been fully completed and an accession number has not yet been assigned.

^†^Sequences generated using 454 technology representing a large number of contigs. These are almost certainly not complete.

^‡^These genes were predicted using EasyGene version 1.2. All other genes were predicted using EasyGene version 1.0.

**Figure 2:**
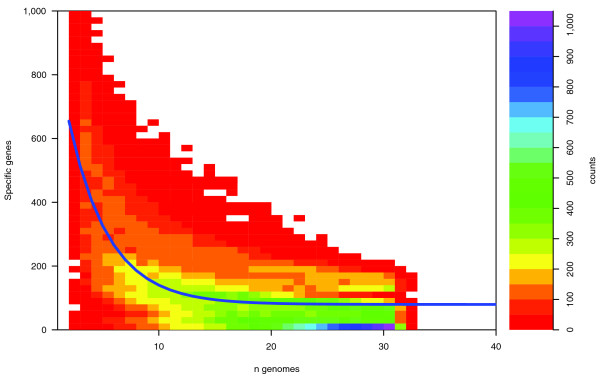
Two-dimensional density plot of novel genome 'specific genes' for the *E. coli *pan-genome. The plot illustrates the number of novel genome specific genes for the nth genome when comparing *n *= 2,...,32 genomes (for a maximum of 3,200 random combinations at each n). The density colors reflect the count of combinations giving rise to a certain number of specific genes (y-axis) in one genome compared to n - 1 other genomes; that is, for *n *= 2, genome number 2 is compared to genome number 1 and, on average, approximately 650 genes are found to be specific to strain 2. The blue line is the fit to the originally suggested exponential decay function [3].

We next estimated how many additional 'strain-specific' genes would be added to the core genome with each genome being sequenced. In this case the decay function defined by [3] was found to be appropriate, as shown in Figure 2. By fitting the data to the number of sequenced genomes approaching infinity, we predict that additional genomes will continue to add approximately 79 genes to the *E. coli *pan-genome, on average. Exploiting the fitted parameters for the data set, the size of the current *E. coli *core genome conserved within the 32 strains included in this study was estimated to contain 2,241 common genes. The estimated size of the current pan-genome was estimated to contain 9,433 different genes. The number of *E. coli *strains used for these estimates is approximately the same as the number of strains present in the human gut [9,10]; thus, the number of *E. coli *genes in the human gut is roughly a third of the number of human genes.

In designing the *E. coli *pan-genome microarray, genes were grouped based on their nucleotide sequences since the probes are based on DNA oligonucleotides. Moreover, the parameters to group genes for similarity were adapted compared to the parameters used for protein similarity to define the core and pan-genome in order to improve differentiation between the nucleotide sequences of similar *E. coli *genes found in different strains. For this purposes the '50% sequence similarity of 50% of the sequence' conservation criteria [3] was found to be sub-optimal. Instead, genes were grouped into gene groups with a slightly different and somewhat stricter homology criteria (see Materials and methods for details), producing a higher number of groupings. This resulted in a total of 11,872 gene groups present in all 32 genomes, compared to the smaller pan-genome of 9,433 gene groups resulting from comparison at the protein sequence level. Of the 11,872 gene groups, 2,041 consisted of genes found in all 32 strains. Thus, the stricter grouping criteria applied here produced a lower number than the currently estimated core genome size of 2,241 protein gene groups for 32 *E. coli *genomes.

In the presented design strategy, the inclusion of 32 *E. coli *strains in the microarray design necessitated the employment of a common standardized gene prediction strategy since some of the genomic sequences had poor or non-existing gene annotations. One option is to either include as many open reading frames as possible as potential genes (in a 'more is better' strategy) or, alternatively, to use EasyGene, a well performing and conservative gene predictor. One can argue that a 'more is better' strategy is preferred to the conservative gene prediction so that fewer genes would be missed. However, including spurious hypothetical genes in the design would potentially obstruct the probe design phase both in the grouping of gene families and in excluding otherwise perfect probes due to cross-hybridization to these false genes. Furthermore, in case of prediction of gene content in control and novel strains by hybridizing genomic DNA to the array, such false positives are just as unwelcome as false negatives. Nonetheless, absence of too many important *E. coli *genes is not desirable either. We therefore compared the genes predicted by EasyGene with the high-quality annotation of the K-12 MG1655 strain (version U00096.3). This revealed that of the 238 protein encoding genes not predicted by EasyGene, 206 were hypothetical genes, leader peptides, frameshifts, gene fragments or pseudogenes. Of the remaining 32 genes, 12 were present in at least one other *E. coli *strain considered in the design. Consequently, only 20 genes of potential interest were missed by EasyGene. Since this is less than half a percent of the genome (20/4,331 = 0.46%), we considered that the advantages of conservative standardized gene finding outweighed the disadvantages of missing a small minority of genes.

### Benchmarking the chip design

**Figure 3:**
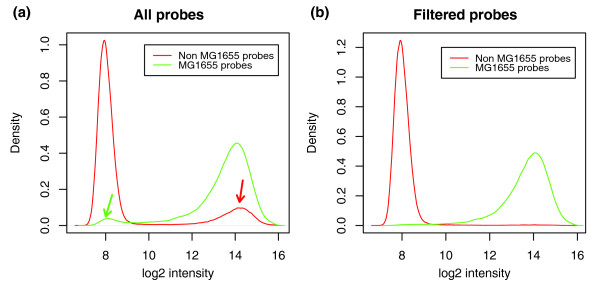
Density plots of probe intensities before and after a filtering step. The density distributions are illustrated for MG1655 probes and non-MG1655 probes separately. Log_2 _intensity data are from a representative MG1655 control sample. **(a) **Before filtering, all probes are divided into MG1655 probes (green lines) and non-MG1655 probes (red lines). It is clear that many probes initially designed for groups containing MG1655 genes do not hybridize well to these, resulting in low intensity (green arrow). Conversely, probes initially designed for groups without MG1655 genes cross-hybridize as if present in MG1655 (red arrow). **(b) **After filtering probes, the remaining probes have the expected hybridization profile.

A pan-genomic approach represents a challenge in evaluating and defining the trade-off in group inclusion stringency: a similarity cut-off chosen too high will result in too many groups, while a low similarity cut-off results in too much sequence variability within a group (producing low conservation scores). Consequently, too much sequence variability within groups will result in group-specific probes producing too low a signal for that group in particular strains. On the other hand, dividing the groups further to limit this undesired inter-group variability causes another problem: some probes may no longer be group specific, leading to undesired cross-hybridization, while other probes might still provide a signal specific for such a group. In the attempt to circumvent these problems, an additional filter step was introduced in the probe design strategy, where probes were removed from further analysis if they were not specific enough to one group and if they did not share a sequence overlap above a certain threshold with the sequences in the group it was designed for (for details refer to Materials and methods). Figure 3a gives an example of how such probes may result in misleading signals, while the signal improves remarkably following exclusion of such probes from the analysis by a filtering step (Figure 3b).

**Figure 4:**
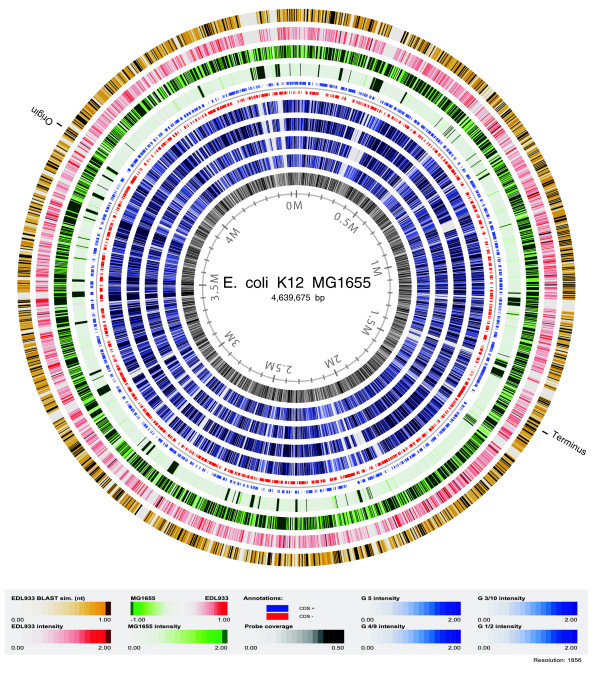
Hybridization and blast atlas. The atlas illustrates the hybridization pattern of MG1655 probes for the two control strains, MG1655 and EDL933, and the four Symbioflor2 isolates. Also, it illustrates the MG1655 genes' BLAST score for presence in the EDL933 strain. The circles from outermost to innermost are: Blast score between 0 for absent and 1 for present MG1655 genes in the EDL933 strain, log_2 _transformed hybridization intensities for EDL933 and MG1655 samples, log_2 _ratio of EDL933/MG1655 samples, location of predicted coding sequences (CDS), log_2 _hybridization intensities for the four Symbioflor2 isolates G5, G4/9, G3/10, G1/2, probe coverage. A zoomable version of the atlas is available at [33].

The chip design was assessed by analyzing and comparing the hybridization data from the two sequenced control strains, EDL933 and MG1655. Both log_2 _intensities and log_2 _ratios were considered. These results are visualized in a hybridization atlas (Figure 4). Here, the median log_2 _intensity and log_2 _ratios of both control strains are illustrated for MG1655 probes, as well as probe coverage for this strain and the sequence similarity at the DNA level of EDL933 genes to MG1655 genes based on BLAST scores. The similarity of the MG1655 probe hybridization pattern for EDL933 to the sequence similarity based on BLAST scores confirms that the probes reflect true biology. The same information is illustrated in the ratio circle (fourth outermost circle), where MG1655 regions absent in the EDL933 genome are clearly visible and correspond to the regions missing in the EDL933 sample (first and second outermost circle). On the contrary, the MG1655 hybridization pattern (third outermost circle) corresponds very well to the probe coverage pattern (innermost circle).

**Figure 5:**
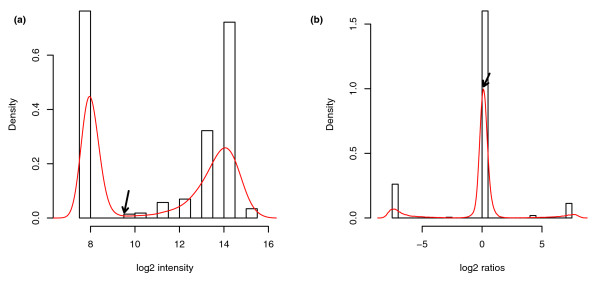
Density distribution histograms. **(a) **Example of bimodal density distribution of log_2 _intensities and histogram of merged log_2 _intensities. The merged level with fewest segments assigned to it is chosen as the cutoff value. All segments with merged values above this cutoff are predicted as present. An arrow indicates the cutoff level for this particular sample. **(b) **Example of unimodal (or trimodal) density distribution of log_2 _ratios and histogram of merged ratios. The merged level with the most segments assigned to it was chosen as the present level. All segments with this merged value or above were predicted as present. An arrow indicates the minimum log_2 _ratio for present probes for this particular sample.

For further analysis, the probes were mapped to each gene group according to the design, and a position-dependent segmentation algorithm was employed to partition data points into present and absent sequence segments [11]. Segmentation was followed by merging the output with MergeLevels [12]. Since the distribution of log_2 _intensities is bimodal - that is, composed of two density distributions (Figure 5a) - it is likely that the best separation of present and absent probes can be found at the local minimum between the two distributions. Consequently, following noise reduction by segmentation and merging, the cutoff for log_2 _intensities was found at the merged value between these two distribution maxima with the least segments assigned to it. All segments with merged values above this cutoff were predicted as present. On the other hand, the distribution of log_2 _ratios is largely unimodal (although two extra, weaker modals occur) (Figure 5b). Since ratios are only calculated for genes present in the control sample, and given the likely high similarity between a test sample and control sample of the same species, most genes are assumed present. Consequently, here the present level was estimated as the merged level to which most segments had been assigned.

Following the filtering step, several gene groups were left with only few probes targeting them, and we found it necessary to remove groups that were targeted by three or fewer probes from further analysis. This reduced the average number of false positives from 267 to 87 (for MG1655) and from 638 to 405 when analyzing all control samples with regard to genes found to be present from analysis of log_2 _hybridization signals compared to genes predicted present from the genome sequence. On the other hand, gene groups represented by few probes were not as likely to result in false negatives since removal of these groups did not change the average number of false negatives significantly (data not shown).

Table 2 lists the resulting sensitivity and false discovery rate (FDR) for all control samples. A very high sensitivity was obtained for both strains, but false positives were suspiciously high for EDL933 (Table 2). For both control strains, a large proportion of the false positive gene groups were consistently identified in replicate samples (a total of 62 and 360 in MG1655 and EDL933, respectively). For MG1655, genes annotated as hypothetical were highly overrepresented among the false positive genes (*P *value approximately 0.002, Fischer's exact test), indicating a significant enrichment in hypothetical genes among false positives. In the majority of cases, the corresponding consensus sequences aligned very well to the genome sequence (with >50% of the sequence length and >91% identity). Consequently, these false positives are not a result of cross-hybridizations but rather a result of genes not predicted by the EasyGene gene finder. Since most of these are seemingly hypothetical and, therefore, are likely not to be real genes, the consequences in terms of strain characterization are considered to be minor.

**Table 2:** Sensitivity and false discovery rate based on analysis of log_2 _intensities

MG1655			EDL933		
	
Chip ID	Sensitivity	FDR	Chip ID	Sensitivity	FDR
108276	0.988	0.021	1004602	0.994	0.13
108667	0.964	0.024	113504	0.988	0.12
113756	0.997	0.021	113509	0.980	0.12
114782	0.999	0.017	113757	0.989	0.13
1509502	0.999	0.043	1509502	0.970	0.11
1510802	0.999	0.015	1510802	0.994	0.11
**Average**	**0.989**	**0.024**	**Average**	**0.986**	**0.12**

Analysis of the hybridization data obtained with MG1655 and EDL933 DNA in six replicates, with data analyzed based on log_2 _intensities. The sensitivity and false discovery rate (FDR) are given for the prediction of gene presence in MG1655 or EDL933 in the corresponding samples.

In contrast to the MG1655 control strain, we did not observe enrichment in hypothetical genes among false positives for EDL933. In this case we suspect that the 'false positives' were actually true genes mistakenly missed by EasyGene. In support of this, EasyGene did actually predict only 4,664 genes for the EDL933 main chromosome compared to the 5,349 annotated in GenBank, possibly due to a number of unknown nucleotides still present in the published genome sequence [13]. Gene expression profiling of these genes would confirm if these are in fact true genes that are expressed and thus incorrectly missed by EasyGene. Preliminary data from a gene expression study run in parallel with this work demonstrated that the gene expression profile of these genes indeed resembled that of other genes present in the EDL933 genome (Sekse C, Friis C, Wasteson Y, Ussery DW and Willenbrock H, unpublished results). This observation supports our interpretation that they are actually not false positives generated by bad chip manufacturing, hybridization artifacts or poor analysis approaches, but a consequence of an ambiguous DNA sequence that any gene predictor would have ignored. Ideally, they should have been categorized as true positives. Consequently, the low FDR obtained from the other control strain, MG1655, is a better indicator of our pan-genome chip performance.

Table 3 compares the performance obtained by analyzing log_2 _ratios of control sample co-hybridizations with the performance based on log_2 _intensities. In both cases, the sensitivity is quite high, while FDR is low, in particular for MG1655. The higher FDR for EDL933 may be assigned to a low accuracy for the gene predictor on this particular genome, as discussed above. While the sensitivity is slightly higher when analyzing log_2 _ratios, FDR is marginally lower when analyzing log_2 _intensities. Consequently, the single channel log_2 _intensity analysis approach offers an acceptable performance compared to the comparative dual channel approach, at a limited risk of increased false negatives but with the added advantage of being able to identify the presence and absence of any gene on the microarray and not only genes present in the control strain.

**Table 3:** Log_2 _intensity results versus log_2 _ratio results for test samples MG1655 and EDL933

	log_2 _intensities		log_2 _ratios	
		
	MG1655	EDL933	MG1655	EDL933
Sensitivity	0.99	0.97	1.00	1.00
FDR	0.003	0.060	0.007	0.063

The sensitivity and false discovery rate (FDR) were compared for data analysis based on log_2 _intensities and log_2 _ratios for the detection of genes in the two control strains for which gene presence is known from gene finding based on the known genome sequence. Thus, only known control gene groups were considered. Consequently, true positives make up the control genes correctly found to be present in all MG1655 or EDL933 samples, respectively. False positives are genes not found in the control strain, but predicted as present from the genome sequence.

### Analysis of probiotic *E. coli *strains

The chip design was next tested for suitability to characterize isolates of non-pathogenic *E. coli *strains. Four probiotic isolates were co-hybridized with MG1655 and EDL933 according to the combinations listed in Table 4; their hybridization pattern to MG1655 probes is illustrated in a hybridization atlas (Figure 4). Here, larger regions absent from the probiotic isolates in comparison to MG1655 are visible. It is also evident that each isolate is different from the next, since each isolate has a distinct hybridization pattern.

**Table 4:** Co-hybridization setup

Chip ID	Cy3 (test)	Cy5 (control)
113756	G 1/2	MG1655
108667	G 3/10	MG1655
114782	G 4/9	MG1655
108276	G5	MG1655
113509	G 1/2	EDL933
113504	G 3/10	EDL933
113757	G5	EDL933
1004602	G 4/9	EDL933
1509502	EDL933	MG1655
1510802	EDL933	MG1655

**Figure 6:**
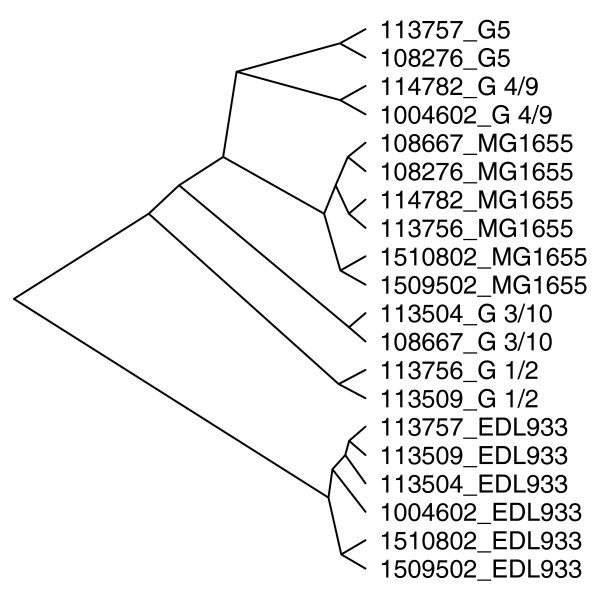
Hierarchical cluster analysis of hybridization signals from the samples summarized in Table 4, including control samples. The analysis is based on remaining probes (refer to Materials and methods for details) after filtering and removal of probes in gene groups with three or less probes. For clustering, the '1-pearson correlation' distance metric was used.

**Figure 7:**
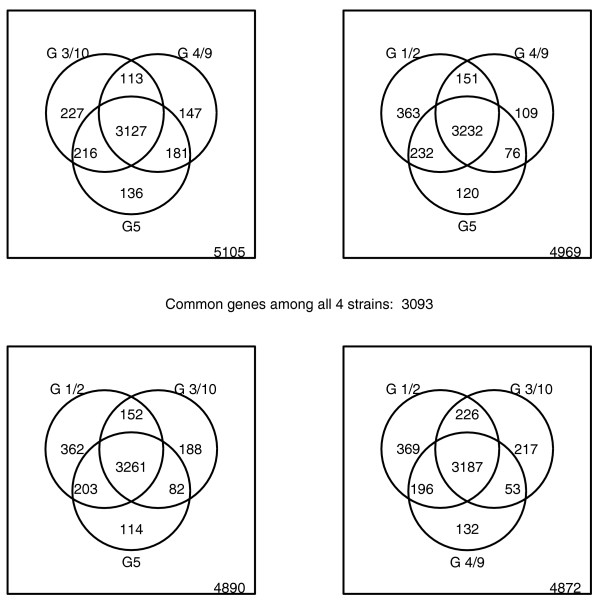
Venn diagram comparing Symbioflor2 isolates.

The gene content of each probiotic isolate was predicted by the single-channel approach as found to be appropriate for this type of analysis. Thereby, the presence of all genes included on the pan-genome array could be assessed for all four test isolates. First, we compared the findings between the isolates used for hybridization. The number of identified genes was highest for G1/2 and lowest for G4/9 (Table 5). Two graphical representations further illustrate the results. Figure 6 shows a cluster analysis based on all probes considered in this paper. The four probiotic isolates cluster individually and form a super-cluster with MG1655 samples, separated from EDL933. Indeed, each isolate shared more of their predicted genes with MG1665 than with EDL933 (Table 5). Moreover, strain-specific genes were more frequently different to EDL933 than to MG1655. This is not surprising since the probiotic isolates are likely to be more related to the non-pathogenic commensal K-12 than to enterohemorrhagic EDL933. Each strain had more than 100 genes that were neither found in MG1655 nor EDL933 (Table 5). Moreover, a significant enrichment was observed in hypothetical genes among the gene groups only found in a single Symbioflor2 isolate. However, this is expected, since *E. coli *core genes are generally better characterized than genes found in only few *E. coli *strains. Figure 7 compares the numbers of genes found to be either unique or shared between one or more probiotic isolates in a Venn diagram. A total of 3,093 genes were found consistently in all four isolates. Figure 6 and Figure 7 both identify isolate G1/2 as the most distantly related to the other isolates.

**Table 5:** Comparison of Symbioflor2 isolates to predictions for control strain samples

	G 1/2	G 3/10	G 4/9	G5
No. of predicted genes	3,978	3,683	3,568	3,660
				
No. of genes in common with (based on log_2 _intensities):				
MG1655	3,464	3,323	3,319	3,399
EDL933	3,455	3,264	3,186	3,237
				
'Novel' sample genes not in (based on log_2 _intensities):				
MG1655	358	251	162	197
EDL933	631	647	635	592
Either control	185	197	126	144

Results are based on log_2 _intensity analyses.

**Figure 8:**
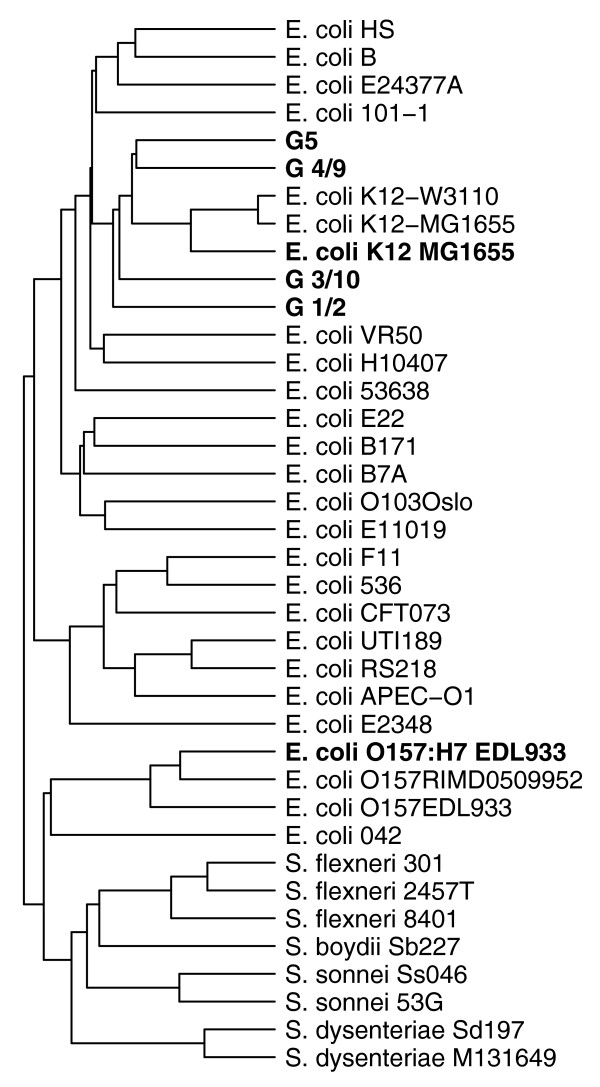
Hierarchical cluster analysis of the 32 design genomes according to gene absence or presence based on the design (normal font). The four Symbioflor2 isolates as well as the two control strains are included in the dendrogram (bold font) by clustering them according to gene predictions based on the sample hybridizations. For clustering, the Euclidian distance metric was used.

Next, genes detected in the probiotic isolates were compared to the genes present (by gene prediction based on their genome sequence) in each *E. coli *strain represented by the chip. All four probiotic isolates shared the most genes with *E. coli *H10407, closely followed by the two K-12 strains for three of the isolates and the VR50 strain for G1/2 (refer to Table S1 in Additional data file 1 for a ranked list of the number of shared genes with the strains considered for chip design). While *E. coli *VR50 is an asymptomatic inhabitant of the urinary tract [14], *E. coli *H10407 is an enterotoxigenic strain. However, its virulence is mostly encoded by plasmids that have not yet been sequenced and, therefore, were not considered in this comparison. Nonetheless, by gene prediction based on the genomic sequence of the H10407 main chromosome, we identified the presence of genes encoding hemolysin (*hly*CABD). These genes were present in probiotic isolate G1/2 as well, in accordance with its weak hemolytic phenotype (described as alpha hemolysis type II; L Beutin and K Zimmermann, unpublished results). Presence of this gene cluster is, however, not sufficient to characterize an isolate as pathogenic [15-17]. Also, the main chromosome of the H10407 strain has previously been found to be highly homologous to *E. coli *K-12 in contrast to other *E. coli *pathogenic strains [18]. This indicates that in spite of the many genes shared with a pathogenic *E. coli *strain, the probiotic isolates are likely to share only the non-virulent parts. Besides, the probiotic isolate shares only marginally more genes with the H10407 strain than with the two K-12 strains (16-57 genes). This is not significant, especially since novel strains are much more likely to share more genes with the large H10407 genome than with the smaller K-12 genomes without actually resembling it more, simply because the H10407 genome encodes 20% more genes. Supporting this, a cluster analysis considering the presence and absence of all gene groups analyzed from our pan-genome array (Figure 8) clearly shows that the gene content of the probiotic isolates is, in fact, more closely related to the gene content of other non-pathogenic strains. In this analysis, all probiotic isolates cluster together with the two K-12 strains while forming a super-cluster with all the other non-pathogenic strains considered in the analysis. This super-cluster contains only a few pathogenic strains for which the sequences of their virulence plasmids were not included in the analysis (strains 101-1, E24377A, and H10407). Furthermore, the validity of the clustering is confirmed by the placement of the control strain MG1655 closest to the two K-12 design strains, and EDL933 closest to the O157:H7 design strains.

Apart from the hemolysin genes and a gene annotated as 'putative iron-regulated outer membrane viruence gene', no other virulence genes were detected in the probiotic isolates. The observed genetic relatedness of probiotic strains to a virulent strain illustrates that both pathogenic and non-pathogenic *E. coli *strains use common strategies for adaptation to their niche. Of the genes found to be present in the probiotic isolates but not in a non-pathogenic *E. coli *strain (MG1655), many were bacteriophage-derived. Nevertheless, complete prophages were not present and variation between and within phage gene content between the four probiotic isolates suggested these bacteriophages have been introduced in independent events. Transposases and other insertion sequence-related genes provided further evidence of the influence of mobile DNA on introducing genetic variation in a bacterial population. Of interest were genes present in the probiotic isolates but absent in MG1655 that were annotated as having general metabolic functions. A closer analysis of these findings would be necessary to assess if such genes provide improved fitness for colonization of the human gut, and so could explain the probiotic nature of the isolates. Also, one should keep in mind that the K-12 isolates represent a reduced *E. coli *genome, and some essential metabolic genes are known to be missing in these isolates. Complete lists of annotated genes found in each of the four Symbioflor2 isolates but not in the MG1655 control strain is provided as Additional data file 2.

## Discussion

The design of a microarray covering more than 30 genomes proved to be a considerable challenge. Multiple aspects had to be considered but the greatest difficulty was to filter out false positives, at the risk of introducing additional false negatives. The level of similarity between gene sequences should justify conserved annotation, but the borders of significance are diffuse and poorly defined. This is a consequence of biological processes that undergo gradual genetic changes. On one hand, probes should cover all versions of the same gene, but at the same time they should be able to distinguish between different genes and even, when relevant, distinct versions of the same gene in different strains. In light of this, conventional microarray design strategies, such as inclusion of mismatch probes for background estimation, will not work when dealing with multiple genomes. One can never ensure that a perfect match is absent for such probes in novel strains. Moreover, because the array should be able to interrogate for the presence of genes at the DNA level (as presented in this paper), the number of probes per gene should be allowed to vary. A higher number of probes is required for a sufficient coverage of long genes, whereas low quality probes would result if attempting to design the same number of probes for very short genes. Consequently, the challenge is to define, in a sensible way, such goals and to search for the best possible solution. Our pan-genome approach proved to be a suitable solution.

Recently, the idea of an 'open pan-genome' was introduced, where each newly sequenced strain would continue to add novel genes to the pan-genome of the species. It was suggested that *Streptococcus agalactiae *would have an open pan-genome, with the consequence that despite sequencing hundreds of strains, novel genes would still be discovered [3]. *E. coli *is likely to also have an open pan-genome since it colonizes multiple environments, complex microflora biotopes, and, therefore, has multiple ways and sources for exchanging and obtaining genetic material [4]. In line with this expectation, Chen and co-workers [19] predicted that each new *E. coli *genome would add 441 genes to the *E. coli *pan-genome pool. However, this prediction of 'new genes' is possibly too high, since it was based on seven very diverse *E. coli *genomes only. Genome size differs considerably within the species, from the relatively small K-12 strains to the larger pathogenic O157:H7 strains. Therefore, we believe that our estimate of the *E. coli *pan-genome and the core genome is closer to what might be expected in the 'real world', since it is based on a much larger number and variety of strains. Thus, the number of added new genes per genome has dropped to about 79 genes when including data from 32 different strains, and may decrease further with improved genome annotations. This smaller estimate is in the same order of magnitude as predicted for other pan-genomes, such as *Streptococcus *(27 per genome for group A and 33 for group B) [3]. Still, our estimate for *E. coli *may be too conservative if the true diversity of *E. coli *is still insufficiently covered by the current genome sequences, that is, environmental and non-mammalian strains are under-represented, and the addition of these may initially add a significant number of novel genes to the *E. coli *pan-genome.

Furthermore, we were able to come up with a more accurate prediction of the *E. coli *core genome. Previously, the size of the *E. coli *core genome was assessed, based on seven different *E. coli *strains, to consist of between 2,865 and 3,475 genes [2,19]. Based on the 32 genomes included in this study, we predict that the size of the core genome will approach approximately 1,560 essential genes, about half that of the previous estimates. We believe the current estimate to be more accurate, as it is based on a much larger number of genomes. However, in the present study, several unfinished genome sequences were included. Improving these both in terms of sequencing and assembly and in gene annotation quality, may result in an increased core genome size if the current partly finished genome sequences are missing core genes.

To assess the performance of the chip as well as to identify the best way of analyzing data from it, control sample hybridizations were analyzed. Comparative hybridizations on dual channel microarrays have the advantage of reduced noise due to limited variations of probe hybridization efficiencies. However, a dual channel analysis is limited to probes covering the control sample so that noise reduction applies only to probes hybridizing to genes present in the control sample. Although the false positive rate was slightly higher for the single-channel analysis approach, we demonstrate that sensitivity is only marginally lower than for the dual channel approach while information can also be extracted regarding genes not present in the control sample. Consequently, this analysis approach offers a favorable possibility for deriving predictions for any gene present on the pan-genome microarray.

Pathogenic *E. coli *genomes are highly overrepresented on this pan-genome chip because the majority of the *E. coli *genomes sequenced to date are from pathogenic strains, and few originate from environmental sources or are commensal strains. Nonetheless, we found that the chip was widely useful for characterizing the gene content of non-pathogenic *E. coli *isolates and for investigating the non-pathogenic nature of these *E. coli *isolates.

Lessons learned from this microarray can be used to design better arrays in the future. Although we considered all designed probes for the chip, including probes with low specificity to all strains in a given gene group, based on our analysis of experimental results, we have found that a filtering step is necessary to remove less specific probes. Moreover, gene groups for which only few probes could be designed (above the probe score cutoff) were not as reliable as gene groups represented by a larger number of probes. While this is not surprising, it nonetheless makes it a difficult task to accurately probe for these genes.

## Conclusion

Based on 32 *E. coli *and *Shigella *genome sequences, we have developed an *E. coli *pan-genome microarray representing the current pan-genome of *E. coli*. Although any individual *E. coli *genome contains between 4,000 and 5,000 genes, we find about twice as many distinct gene groups in the total gene pool examined. High-density pan-genome microarrays can be quite useful for characterizing either DNA content or gene expression from unknown *E. coli *strains. Thus, we found the technique highly sufficient to investigate gene content of four non-pathogenic *E. coli *isolates despite the strong bias for pathogenic strains represented on the pan-genome array. The four analyzed probiotic *E. coli *isolates share a gene pool very similar to the *E. coli *K-12 strains, and additional strain-specific genes were often phage genes, transposases, insertion elements and metabolic genes. It remains to be seen to what degree these genes contribute to the probiotic nature of the isolates. Generally, we conclude that our high-density pan-genome array provides an excellent tool for characterizing the genetic makeup of unknown *E. coli *strains and can also deliver insights into phylogenetic relationships.

## Materials and methods

Twenty-four *E. coli *chromosome sequences that were publicly available at the time of analysis (as one or multiple contigs) and nine plasmid sequences belonging to seven of the sequenced strains were included in this study. In addition, eight *Shigella *chromosomes were included (two *S. sonnei*, three *S. flexneri*, two *S. dysenteriae *and one *S. boydii*) with their three corresponding plasmids (Table 1).

### Probe and microarray design

All considered genome and plasmid sequences (Table 1) were searched for genes using EasyGene version 1.0 or 1.2 [7,8] in order to standardize gene finding. Genes were screened for homology using BLAST [20] in order to prevent redundancy of the probes. Genes were placed in a group when homologous by the following criteria: E-value <10^-5^, bitscore >55, and alignment constituting 50% or more of the longer of the two aligned sequences. Genes with no homology were represented as 'singles'. Groups of genes ('multiples') were aligned using ClustalW with default settings [21] and a consensus sequence was derived using the most frequent nucleotide at each position, weighted by its background frequency in all genes. The probe design strategy employed by OligoWiz [22] was used for probe selection. Two additional scores were introduced as parameters for the probe design software dealing with consensus sequences: a weighted conservation score and a gap score. The weighted conservation score uses Shannon's information measure [23] for conservation at each nucleotide position in a probe. According to [24], the influence of a mismatch on measured hybridization intensities varies with its position, with positions towards either end having less influence. Therefore, each position was weighted according to a second order polynomial function. The probe's weighted conservation score is the product of the weighted position scores for mismatch basepairs. The gap score was used to identify probes that targeted gaps in the alignment of multiples. This score was used to design probes that specifically identified conserved regions of all genes in each group (thus attempting to avoid gaps).

All probes were designed as 55-60 mers, with variable length and sequence to optimize for the same melting temperature. Only standard nucleotides (GATC) were considered in the probe design. In total, 305,285 probes covering 11,768 gene groups and singles were designed. A detailed description of the microarray design may be found in Additional data file 3. The probe design was given the NimbleGen design_id 5137 and is available upon request.

### Filtering of probes

Probes were aligned against all predicted gene sequences included using NCBI-BLAST blastn version 2.2.11 [25] and the identity of each probe with each gene sequence was determined in base pairs. Sequences were extracted for which the ratio [bp identity/probe length] was >0.8. Probes that either matched more than one single group or failed to match all genes in the design group were excluded from further analysis. Furthermore, groups for which three or less probes remained after filtering were removed from the subsequent analysis due to their increased risk of generating false positives (see Results). This resulted in a reduction to 224,805 probes covering 9,252 gene groups and singles. Consequently, the number of probes targeting each gene ranged from 4 to 29 with a median coverage of 27 probes per gene.

### Annotation of gene groups

Each gene group in the probe design was annotated according to the results from hits against the UniProtKB/Swiss-Prot release 52.5 and UniProtKB/TrEMBL release 35.5 protein database [26] using NCBI-BLAST Blastp version 2.2.11 [25]. Only alignments covering >50% of the gene lengths and having 50% or better identity within the alignment were included. Among all the sequences within each group, the hit producing the highest percent identity was chosen. In this way, 5,348 of our 11,872 gene groups could be annotated against Swiss-Prot and 9,320 of our 11,872 gene groups could be annotated against TrEMBL. Thus, while Swiss-Prot generally produces more reliable annotations, the number of annotations produced was quite low. Consequently, when available, genes were assigned the more reliable Swiss-Prot annotation, otherwise it was assigned the TrEMBL annotation if one was available. Gene groups that could not be assigned an annotation were assigned hypothetical proteins.

### Pan-genomics

The pan-genome was estimated as suggested by Tettelin *et al. *[3], with modifications to reduce computational load for our large dataset. Briefly, protein sequences were compared by Blastp version 2.2.11 [25]. Proteins with at least 50% sequence identity over at least 50% of their length were identified as the same. For each n additional genome, genome n was compared to any combinations of n - 1 genomes, and the number of identical 'core genes' and 'strain-specific genes' (specific for strain n) were counted for each n. According to the approach suggested, when all genomes are compared to n other genomes (*n *= 1,..,N), this would result in 32!/[(*n *- 1)!·(32 - *n*)!] possible combinations (for each n) of drawing n genomes out of the pool of 32 genomes. Consequently, for 16 or 17 genomes (*n *= 17,18 in above formula), a total of 9.62 billion possible combinations exists. To reduce computational time, we lowered the number of combinations by randomly selecting 3,200 different combinations (or the maximum number of combinations; 3,200 is dividable by 32, which ensures that all genomes are used an equal number of times as blast template for each n) with an equal distribution among query genome. This was repeated ten times and an exponential decay function was fitted to each of these repeats. The decay function suggested by Tettelin *et al *[3] was first applied:

F(n)=κ⋅e(−nτ)+g

where *g *equals the number of 'core genes' or 'specific genes', while κ and τ are free parameters for amplitude of exponential decay. The speed at which F(n) converges was found to fit the data for 'strain-specific genes' satisfactorily, while a modified decay function with the double square root of n was found to fit the 'core genes' data better (lower sum of squared errors):

F(n)=κ⋅e(−sqrt(sqrt(n))τ)+g

### Strain selection, DNA preparation and hybridization

Control strain K-12 MG1655 was kindly provided by Flemming G Hansen (CBS, BioCentrum-DTU, The Technical University of Denmark) and genomic DNA from control strain O157:H7 EDL933 was kindly provided by Camilla Sekse (Norwegian Veterinary school, Oslo). As test strains, Kurt Zimmermann from Symbiopharm (Herborn, Germany) supplied four probiotic *E. coli *isolates, designated G1/2, G3/10, G4/9 and G5, from their commercially available Symbioflor2 product. G1/2 has previously been serotyped as O rough:K-:H- and O rough:H-, G3/10 as O 35,129:K-:H-, G4/9 as O rough:K-:H-, and G5 as O rough:K-:H-.

All test strains were grown overnight in Luria-Bertani (LB) broth with continuous agitation [27], and DNA was isolated as described previously [28]. The genomic DNA was labeled with cy3 or cy5 and hybridized to NimbleGen custom arrays according to NimbleGen standard protocols for CGH (prepared and hybridized by NimbleGen (Madison, Wisconsin USA)). The raw data are available from the Gene Expression Omnibus (GEO) database [29] with series accession number GSE8595.

### Analysis methods

The probes were mapped to each gene group including position according to the design and analyzed as described previously [2] with minor modifications. Briefly, a position-dependent segmentation algorithm was employed to partition data points into present and absent sequence segments constituting any given gene. For this, we used circular binary segmentation [11] with default settings as implemented in DNAcopy developmental version 1.2.1 written for the R statistical language [30]. As recommended by the authors, the data were first smoothed and subsequently segmented. Segmentation was followed by merging the output with MergeLevels [12] with a fixed threshold at the standard deviation between segmented log_2 _intensities and observed log_2 _intensities, or the standard deviation of segmented log_2 _ratios.

Consequently, following noise reduction by segmentation and merging, the cutoff for log_2 _intensities was found at the merged value between these two distribution maxima with the least segments assigned to it. All segments with merged values above this cutoff were predicted as present. Since ratios are calculated only for genes present in the control sample, and given the likely high similarity between a test sample and control sample of the same species, most genes are assumed present. Consequently, here the present level was estimated as the merged level to which most segments had been assigned. Moreover, for a gene to be called present, at least 90% of its probes should be found to be present. Accordingly, the samples were both analyzed individually as log_2 _intensities and combined with the appropriate control experiment, as log_2 _ratios.

Atlases were created using the GeneWiz software [31]. The blast atlases were constructed as described previously [32].

## Abbreviations

aCGH, comparative genomic hybridization; FDR, false discovery rate.

## Authors' contributions

HW and PFH designed the microarray. HW performed experimental work, analyzed the data and drafted the manuscript. DWU collected the genome sequences and supervised the project. TMW contributed with biological insight into *E. coli *pathogenicity. All authors edited and approved the final manuscript.

## Additional data files

The following additional data are available with the online version of this paper. Additional data file 1 is a table providing a ranked list of each Symbioflor2 isolate's similarity to chip design strains. Additional data file 2 contains complete lists of annotated genes found in each of the four Symbioflor2 isolates but not in the MG1655 control strain. Additional data file 3 contains a detailed description of the microarray design.

## Supplementary Material

Additional data file 1 Ranked list of each Symbioflor2 isolate's similarity to chip design strains. Click here for file

Additional data file 2 Annotated genes found in each of the four Symbioflor2 isolates but not in the MG1655 control strain. Click here for file

Additional data file 3 Detailed description of the microarray design. Click here for file
