# Supplementary material for: Characterization of probiotic Escherichia coli isolates with a novel pan-genome microarray
Source: Genome Biol. 2007 Dec 18;8(12):R267. doi: 10.1186/gb-2007-8-12-r267 (PMC2246269; doi:10.1186/gb-2007-8-12-r267)
Supplement: Additional data file 1 — Ranked list of each Symbioflor2 isolate's similarity to chip design strains. [file gb-2007-8-12-r267-S1.doc]

Table S 1. Ranked list of similarity to chip design strains. Numbers in parentheses are the number of genes predicted in each Symbioflor2 isolate that according to the chip design also exist in each of the chip design strains.

| G 1/2 | G 3/10 | G 4/9 | G5 |
| --- | --- | --- | --- |
| E. coli H10407 (3590)  E. coli VR50 (3553)  E. coli K12-MG1655 (3533)  E. coli K12-W3110 (3533)  E. coli E22 (3495)  E. coli O103Oslo (3489)  E. coli E24377A (3488)  E. coli O157RIMD0509952 (3473)  E. coli B (3455)  E. coli 042 (3453)  E. coli 101-1 (3447)  E. coli E11019 (3435)  E. coli HS (3425)  E. coli 53638 (3412)  E. coli B171 (3390)  E. coli B7A (3369)  E. coli UTI189 (3363)  E. coli APEC-O1 (3358)  E. coli CFT073 (3354)  E. coli RS218 (3309)  E. coli 536 (3308)  S. sonnei 53G (3283)  E. coli E2348 (3278)  E. coli F11 (3278)  S. sonnei Ss046 (3256)  E. coli O157EDL93 (3245)  S. flexneri 2457T (3170)  S. flexneri 8401 (3168)  S. flexneri 301 (3167)  S. boydii Sb227 (3067)  S. dysenteriae Sd197 (2881)  S. dysenteriae M131649 (2877) | E. coli H10407 (3387)  E. coli K12-MG1655 (3368)  E. coli K12-W3110 (3367)  E. coli E24377A (3365)  E. coli HS (3356)  E. coli VR50 (3345)  E. coli O103Oslo (3332)  E. coli E22 (3323)  E. coli B (3310)  E. coli 53638 (3285)  E. coli 042 (3269)  E. coli O157RIMD0509952 (3267)  E. coli 101-1 (3266)  E. coli E11019 (3252)  E. coli B7A (3241)  E. coli B171 (3230)  S. sonnei 53G (3175)  E. coli APEC-O1 (3175)  E. coli UTI189 (3171)  S. sonnei Ss046 (3155)  E. coli CFT073 (3144)  E. coli 536 (3130)  E. coli RS218 (3125)  E. coli E2348 (3113)  S. flexneri 8401 (3113)  E. coli F11 (3100)  S. flexneri 2457T (3088)  S. flexneri 301 (3082)  E. coli O157EDL93 (3052)  S. boydii Sb227 (3006)  S. dysenteriae Sd197 (2810)  S. dysenteriae M131649 (2796) | E. coli H10407 (3352)  E. coli K12-MG1655 (3336)  E. coli K12-W3110 (3336)  E. coli VR50 (3305)  E. coli E24377A (3284)  E. coli B (3276)  E. coli 53638 (3243)  E. coli HS (3239)  E. coli 042 (3235)  E. coli O103Oslo (3218)  E. coli E22 (3216)  E. coli 101-1 (3213)  E. coli O157RIMD0509952 (3184)  E. coli B171 (3150)  E. coli E11019 (3139)  E. coli B7A (3127)  E. coli APEC-O1 (3088)  E. coli UTI189 (3074)  E. coli CFT073 (3072)  E. coli 536 (3069)  S. sonnei 53G (3059)  E. coli E2348 (3043)  S. sonnei Ss046 (3035)  E. coli F11 (3023)  E. coli RS218 (3021)  E. coli O157EDL93 (2976)  S. flexneri 2457T (2962)  S. flexneri 301 (2959)  S. flexneri 8401 (2959)  S. boydii Sb227 (2864)  S. dysenteriae Sd197 (2710)  S. dysenteriae M131649 (2690) | E. coli H10407 (3441)  E. coli K12-MG1655 (3396)  E. coli K12-W3110 (3396)  E. coli VR50 (3348)  E. coli HS (3296)  E. coli E24377A (3294)  E. coli B (3290)  E. coli E22 (3253)  E. coli 101-1 (3250)  E. coli O103Oslo (3242)  E. coli 042 (3235)  E. coli O157RIMD0509952 (3222)  E. coli 53638 (3216)  E. coli E11019 (3193)  E. coli B171 (3192)  E. coli B7A (3175)  E. coli CFT073 (3141)  E. coli APEC-O1 (3139)  E. coli UTI189 (3132)  E. coli RS218 (3117)  E. coli 536 (3103)  E. coli E2348 (3092)  S. sonnei 53G (3087)  E. coli F11 (3067)  S. sonnei Ss046 (3061)  E. coli O157EDL93 (3013)  S. flexneri 8401 (3009)  S. flexneri 2457T (2989)  S. flexneri 301 (2984)  S. boydii Sb227 (2917)  S. dysenteriae Sd197 (2722)  S. dysenteriae M131649 (2706) |
