# Supplementary material for: Characterization of probiotic Escherichia coli isolates with a novel pan-genome microarray
Source: Genome Biol. 2007 Dec 18;8(12):R267. doi: 10.1186/gb-2007-8-12-r267 (PMC2246269; doi:10.1186/gb-2007-8-12-r267)
Supplement: Additional data file 3 — Detailed description of the microarray design. [file gb-2007-8-12-r267-S3.pdf]

Design of *E. coli* chip v2 (pan-genome chip)

Sequences

We have included the following strains in the design, primarily obtained from NCBI GenomeProjects [34]:

Strain	Accession	NCBI Proj ID	contigs	ORFs	length
Escherichia coli 042 chromosome	-[a]	340	1	4607	5 241 977
Escherichia coli 042 plasmid	.	340	1	106	113 346
Escherichia coli 101-1 chromosome	AAMK01000001-70	16193	70	4353	4 880 382
Escherichia coli 53638 chromosome	AAKB01000001-119	15639	119	4779	5 289 471
Escherichia coli 536 chromosome	CP000247	16235	1	4341	4 938 920
Escherichia coli B chromosome	-	18083	1	4076	4 629 819
Escherichia coli B171 chromosome	AAJX01000001-159	15630	159	4780	5 299 753
Escherichia coli B171 plasmid	AB024946	15630	1	69	68 817
Escherichia coli B7A chromosome	AAJT01000001-198	15572	198	4646	5 202 558
Escherichia coli CFT073 chromosome	AE014075	313	1	4653	5 231 428
Escherichia coli E11019 chromosome	AAJW01000001-15	15578	115	4839	5 384 084
Escherichia coli E22 chromosome	AAJV01000001-109	74230453	109	4943	5 516 160
Escherichia coli E2348 chromosome	-	341	4	4592	5 071 653
Escherichia coli E2348 pB171 plasmid	-	341	1	70	68 890
Escherichia coli E2348 p9123 plasmid	-	341	1	5	6 293
Escherichia coli E2348 pGEPAT plasmid	-	341	1	3	2 233
Escherichia coli E24377A chromosome	AAJZ01000001	13960	1	4407	4 980 187
Escherichia coli F11 chromosome	AAJU01000001-88	15576	88	4593	5 206 906
Escherichia coli H10407 chromosome	-	-	89	4865	5 428 706
Escherichia coli HS chromosome	AAJY01000001	13959	1	4126	4 643 538
Escherichia coli K12-MG1655 chromosome	U00096	225	1	4122	4 639 675
Escherichia coli K12-W3110 chromosome	AP009048	16351	1	4133	4 646 332
Escherichia coli O103Oslo chromosome[b]	-	-	1115	4571	5 231 845
E. coli O157RIMD0509952 chromosome	BA000007	226	1	4989[c]	5 498 450
Escherichia coli O157RIMD0509952 pO157	AB011549	226	1	70	92 721
Escherichia coli O157RIMD0509952 pOSAK1	AB011548	226	1	3	3306
Escherichia coli RS218 chromosome	-	-	1	4898	5 089 234
Escherichia coli RS218 plasmid	-	-	1	115	114 233
Escherichia coli UTI189 chromosome	CP000243	16259	1	4466	5 065 741
Escherichia coli UTI189 plasmid	CP000244	16259	1	114	114 230
Escherichia coli VR50 chromosome[b]	-	-	1228	4453	5 064 870
Escherichia coli APEC-O1 chromosome	CP000468	16718	1	4551	5 082 025
Escherichia coli O157EDL933 chromosome	NC_002655	259	1	4664 [c]	5 528 445
Escherichia coli O157EDL933 plasmid	AF074613	259	1	70	92 077
Shigella boydii Sb227 chromosome	CP000036	13146	1	4356	4 519 823
Shigella dysenteriae M131649 chromosome	-	346	234	4755	4 962 690
Shigella dysenteriae Sd197 chromosome	CP000034	13145	1	4237	4 369 232
Shigella dysenteriae Sd197 pSD1197	CP000035	13145	1	160	182 726
Shigella flexneri 2457T chromosome	AE014073	408	1	4388	4 599 354
Shigella flexneri 301 chromosome	AE005674	310	1	4410	4 607 203
Shigella flexneri 301 pCP301 plasmid	AF386526	310	1	194	221 618
Shigella flexneri 8401 chromosome	CP000266	166375	1	4383	4 574 284
Shigella sonnei 53G chromosome	-	-	5	4780	5 220 473
Shigella sonnei Ss046 chromosome	CP000038	13151	1	4443	4 825 265
Shigella sonnei Ss046 pSS plasmid	CP000039	13151	1	179	214 396

[a]The genome sequence has not been completed and an accession number has not yet been assigned.
[b] Sequences generated using 454 technology representing a large number of contigs are almost certainly not complete
[c]These genes where predicted using EasyGene version 1.2. All other genes were predicted using EasyGene version 1.0.

**Segment**	**Count**
E.coli Main chr.	24
Shigella Main chr.	8
E.coli Plasmids	10
Shigella Plasmids	3

Gene prediction

Genes have been predicted using in-house EasyGene version 1.0 with an R-cutoff of 2.0. All coding sequences have been included in the design.

For two of the *E. coli* genomes a program bug has prevented the prediction of genes in both strands and for these two sequences a gene prediction has been downloaded from Copenhagen University [35] - the predictions provided are made using EasyGene 1.2 with 2.0 as R-cutoff.

Blast

In order to reduce the homology between similar genes among the different strains and alignment and consensus approach has been chosen.

All orfs have been BLASTed against each other and labelled “Homologous” by the following criteria:

1. E-value better (lower) than 10^{-5}
2. Score better (higher) than 55 – equivalent of the score of the shortest two sequences.
3. The alignment length should constitute 50% or more of the longer of the two aligned sequences.

All homologous genes are grouped recursively. Being least conservative (disregarding the depth of similarity) genes A, B,C,and D are grouped if A+B and B+C and C+D are homologous, even though A+C has not been marked as homologous by the definitions above. A second step is introduced which removed sequences which are least homolgous to all others. This is done by counting all-against-all matches within the group and sorting by the count. The gene with the least similarity is removed if different from the maximum similarity. After a sequence is removed, the all-against-all similarity is recalculated. This is done until all sequences have matches to all sequences. The second step was included to prevent small sequences from being grouped in the same consensus group if one was similar to the first half of the consensus and the other similar to the end second half of the consensus. If this is the case, the derivation of the consensus is better off excluding these highly homologous short sequences.

Genes with no homology were placed in groups of only the sequence itself (‘singles’) and the groups with more than one sequence (‘multiples’) are aligned using CLUSTALw with standard settings [21].

In each alignment the consensus sequence is derived by weighting the frequency of the nucleotides at residue *r*, with the background frequency in all genes in the group. That is, base *N* is chosen if it has the largest weighted frequency, W_r:

$$W_r = f_{\underline{r},\, N \in A,\, T,\, G,\, C} \times w_N$$

Where $f_{r,N}$ is the frequency of base *N* at the residue *r* and w_N is the background frequency of base *N* in the genes. Gaps are not counted and will not occur in the consensus.

For larger windows having less conservation there exist increased possibility of partial hybridization and cross-hybridization. A score of Shannon information measure is introduced to prioritize probes having higher conservation this is described below.

Weighted conservation scores (between 0 and 1000)

Generally, conservation scores refer to the Shannon's information measure [23]:

$$R_i = 2.0 + \sum_{a=A}^{T} P_i^a \cdot \log_2(P_i^a)$$

This is a value between 0 and 2, where 2 refer to a fully conserved position in an alignment and 0 to a position where either base is equally frequent. Consequently, for a set of perfectly aligned sequences, the conservation score will consists of a row of 2's and the weighted conservation score of probes targeted at this alignment would be 1000.

According to [24], the influence of a mismatch on the measured hybridization intensities varies with its position, with positions in the end having less influence[1]. Therefore, the performance for a probe targeted at a set of non-perfectly aligned sequences will not only depend on the number of the mismatches and their conservation score, but also on the position of the mismatches.
Thus, this influence was modeled by a second order polynomial function depending on the length of the probe:

$$w_i = \frac{1-\sqrt{0.5}}{(1-m)^2} * \left(i-m\right)^2 + sqrt(0.5)$$

Here, w_i is the weight for the ith position, and m is the middle position of the probe (probe length / 2 + 0.5). This gives a parable with minimum value of sqrt(0.5) at the middle bp position and the value of 1 at either end.

The shannon entropy value (R) for each mismatch bp is scaled to a value between sqrt(0.5) and 1.

$$R_{i,scaled} = \frac{E_i}{2} * \left(1-\sqrt{0.5}\right) + \sqrt{0.5}$$, i=1....probe length ith position

The probe's weighted conservation score is the product of the weighted position scores for mismatch bp's.

$$\text{weighted conservation score} = 1000 * \prod_{j}^{N} \mathrm{E_j}$$

Here, j is the indices of any mismatch probes.

Consequently, when position dependent weights are multiplied with shannon entropy values, the following criteria are fulfilled:

A center mismatch position with conservation score 0 would be weighed with sqrt(0.5) (w=0.71) and therefore result in an overall weighted conservation score of 500.

---

[1] Although they found less influence from mismatches at the surface end of the probe than the solution end, we chose to model the influence with equal weight to both ends, and most in the middle, since their experimental measurements where noisy and also specific to the microarray technology used in their study.

An end mismatch position would not be down-weighted (w=1) and a probe with a single end mismatch would receive an overall weighted conservation score corresponding to its scaled shannon conservation value (between sqrt(2) and 1) multiplied by 1000.

Gap score

For each probe targeted at a consensus sequence (a sequence based on an alignment of multiple predicted orfs), a gap score is calculated as the maximum fraction of the probe length that is targeted at a gaps.

gap score = number of gaps / length of probe * 1000

For example, for a probe where the targeted consensus sequence is based on the following alignment, the gap score would be 500.

- - - - ATGC
ATGCATGC

While the gap score was used both as a score to avoid targeting gaps in the alignment as well as a means of distinguishing between sequences from different strains, the inverse gap score was defined as ‘1- gap score’, to give high scores for probes avoiding gap areas.

Probe selection – area 1 and 2

The scores just introduced (conservation score and gapscore) were combined with standard design properties folding, melting, complexity, and homology which are calculated by oligowiz [22].

The homology and complexity databases used by OligoWiz were generated with the format_oligowiz_db backend utility provided by Henrik Bjørn - format_oligowiz_db.pl (available upon request).

Options used:
-genome [consensus fasta file] -dbname “ec02_score55”

For calculating probe properties we used the backend Perl script for OligoWiz2 - generic.oligowiz2.pl (available upon request).

Options used:
-lmin=55 -lmax=60 -length=57 -posscoretype=1 -embed.

Two areas are defined:
Covering the conserved regions (low gap score, high conservation score)
Regions of present absent strectches – typically flanking regions of the genes (high gap score, high conservation score)

The overall scores are calculated by summarizing property *p* weighted by w_p.

$$s_w = \frac{\sum_{p \in A..G} \frac{P_p}{1000} \cdot w_p}{\sum_p w_p}$$

The scores are weighted as follows:

Property (score 0-1000)	*Sub-design 1*	*Sub-design 2*
A. Cross hybridization	2.0	2.0
B. Melting temperature	2.0	2.0
C. Position	(not included)	(not included)
D. Weighted information	2.0 (≥500)	2.0 (≥500)
E. Complexity	0.7	0.7
F. Gap score	(not included)	2.0 (≥500)
G. 1000 – gap score	2.0 (≥500)	(not included)
H. Folding	1.0	1.0

Probes within the individual group are sorted by the weighted score. Starting from the top of this list, probes are selected if: 1) The score is bigger than the minimum score allowed (I) and the distance to the any of the probes selected is smaller than the minimum allowed (K). The minimal score allowed is neglected if the total number of probes designed for the group, is below the minimum (H). The process will stop when the maximum number of probes (H) is reached. All probes including non-standard nucleotides (standard=ATGC) were removed before the probe selection step.

Parameter	*Subdesign 1*	*Subdesign 2*
H. Minimum number of probes (disregarding score)	2.0	2.0
I. Minimum score	0.0 (0.41732 observed)	0.0 (0.390402 observed)
J. At least n probes	10	0
K. Minimum probe distance	10	10
L.Maximum probes pr. gene	29	13
Number of probes	305 285	33 696

Intergenic regions – sub-design 3

Intergenic segments where extracted for *E. coli* K12 MG1655 and *E. coli* O157:H7 EDL933 between genes (not distinguishing between strand). Probes were designed for both the positive and negative strand of each of these segments.

If intergenic segments were longer than 225 bps, they were divided into fragments, for which oligos were targeted in the subsequent design phase, to avoid the close clustering of probes in smaller areas of a longer intergenic stretch. The number of fragments was decided according to the following formula (floor):

$n = \lfloor \text{sqrt(length/100)} \rfloor$

Consequently, there is a non-linear relationship between length and number of fragments at which probes should be targeted, i.e. the number is increasing only moderately with increasing lengths of the intergenic stretches.

OligoWiz v2.1.0 (13th of December , 2005) and a custom script were used to design a maximum of 3 probes for each intergenic segment or fragment with a minimum of 30 bps between start positions and a minimum weighted score of 0.5.
Other settings: min/max/aim oligo length 55/60/57, Tm model: DNA:DNA, position: mittle primed (only for score, not included in overall score). Optimum temperature mean of those for genes (84). All probes including non-standard nucleotides (standard=ATGC) were removed before the probe selection step.

Weighted scores based on the following scores:

Property (score 0-1000)	*Sub-design 3*
A. Cross hybridization	2.0
B. Melting temperature	2.0
C. Position	(not included)
D. Weighted information	(not included)
E. Complexity	0.7
F. Gap score	(not included)
G. 1000 – gap score	(not included)
H. Folding	1

Total: 46998 probes

Truncation

103 probes were truncated from the 3’ end (right) due to the requirement of more than 180 synthesis cycles.

NimbleGen synthesizes 3'->5' in A, C, G, T order. Each synthesis base counts as a cycle. Therefore the least expensive probe to make is TGCA (5'->3') and the most expensive is ACGT.

The probe ids and sequences of the truncated probes may be found in the file ‘probes_truncated.ckd’.

Experimental design

The following Symbioflor2 *E. coli* isolates and control strains were included in this study:

Symbioflor2 isolates	*Date*	*Conc. ug/ul*
S2 G 1/2	21.06.2005	1.4195519
S2 G 3/10	22.06.2005	0.8157120
S2 G 4/9	23.06.2005	1.6990042
S2 G 5	28.06.2005	1.4160642

Control strains	*Date*	*Conc. ug/ul*
K-12 MG1655	21.06.2005	1.7247639
O157:H7 EDL933	22.06.2005	

Dilution schema, NimbleGen requires 250 ng/ul -> 0.25 ug/ul, at least 2.5 ug (4 ug for pathogenic):

	Sample vol	TE buffer	Tot. volume	Total amount
S2 G 1/2	7.04 ul	33 ul	40 ul	10 ug
S2 G 3/10	12.2 ul	27.7 ul	40 ul	10 ug
S2 G 4/9	5.9 ul	34.1 ul	40 ul	10 ug
S2 G 5	7.1 ul	32.9 ul	40 ul	10 ug
K-12 MG1655	11.6 ul	68.4 ul	80 ul	20 ug
O157:H7 EDL933			80 ul	20 ug

Hybridization scheme

8x array CGH with samples in duplicates, but only single copies of each sample/control pair. Additional replicated control strain hybridizations.

Array#	*Test strain*	*Control*
1	S2 G 1/2	K-12 MG1655
2	S2 G 1/2	EDL933
3	S2 G 3/10	K-12 MG1655
4	S2 G 3/10	EDL933
5	S2 G 4/9	K-12 MG1655
6	S2 G 4/9	EDL933
7	S2 G 5	K-12 MG1655
8	S2 G 5	EDL933
9	EDL933	K-12 MG1655
10	EDL933	K-12 MG1655
